# Challenges to Employing Shared Decision Making With Adults Under Community Supervision Who Have a Mental Illness

**DOI:** 10.3389/fpsyt.2021.773411

**Published:** 2021-11-04

**Authors:** Jason Matejkowski

**Affiliations:** School of Social Welfare, University of Kansas, Lawrence, KS, United States

**Keywords:** community corrections, shared decision making, dual role, mental illness, stigma, strengths, parole, probation

## Abstract

Adults under community corrections supervision and who have a mental illness (MI) are expected to comply with conditions of release which often include involvement with supportive social services. The rates of technical violation, arrest, and incarceration that result from failure to comply with these mandates are exceedingly high. Shared decision making among officer-supervisors and client-supervisees is a promising approach to promote engagement in community corrections services among supervisees who have MI. This paper reviews recent research on shared decision making and identifies three barriers to its implementation in this context: (1) a lack of role clarity, (2) a predilection for risk avoidance, and (3) stigma toward supervisees. Empirically supported recommendations are suggested to aid in overcoming these obstacles, facilitate shared decision making, and promote recovery among this population: (1) unification of supervisor rehabilitative and public safety roles, (2) maximizing opportunities for self-determination through low-stakes events and/or enhancement of supervisee strengths and capabilities, and (3) supervisor training in principles of mental health recovery.

## Introduction

People with mental illness (MI) are overrepresented among the nearly 4.4 million adults living under community corrections supervision in the United States [i.e., on probation or parole (1–3)]. In general, persons under community supervision (supervisees) must comply with certain conditions of release and adhere to a range of supervising officer instructions. These supervision requirements may be more demanding for people with MIs as, in addition to the standard conditions required of all supervisees, mandates for these individuals often include participation in mental health or substance use treatment and adherence to the recommendations of these specialty treatment providers. The high rates of arrest and incarceration that result from the failure to adhere to supervision requirements [termed technical violations (4, 5)] suggest that alternative methods to encourage supervisee engagement in supportive treatment services are needed to reduce returns to incarceration. One such approach, that of shared decision making, is promising in the effort to promote engagement in community corrections services among adults who have MI. However, fundamental concerns may serve as an obstacle to advancement of shared decision making in this setting.

Essential elements of collaborative decision making have been advanced since the inception of shared decision making was featured in the medical literature about 50 years ago and they have experienced increased resonance via the recovery movement in mental health care that has been ongoing since the 1990s. In this context, recovery has been defined as “a process of change through which people improve their health and wellness, live self-directed lives, and strive to reach their full potential” (6). As such, services may be considered “recovery-oriented” when they promote self-determination by empowering individuals to have a voice in directing the care they receive and the resources and supports they use to support their treatment and/or rehabilitation (7). Shared decision making supports the recovery process by providing the service structure and practices that allow for the consumer's voice to be heard and empower that voice to influence care and treatment.

Sharing information with the person receiving services, presenting treatment options, understanding the preferences of the person, discussing the risks and benefits of treatments, providing recommendations, and setting forth helpful information about how necessary decisions might be made are all part of shared decision making (8). Recent reviews indicate shared decision making interventions are associated with a number of positive outcomes in general health care (9) and mental health care, including feelings of increased empowerment and reduced coercion among clients in relation to their care (10). In the criminal justice field, features and outcomes of shared decision making align with research on procedural justice and legitimacy theory indicating that justice-involved persons who report being treated fairly, collaboratively, and according to transparent policies and procedures (i.e., in a procedurally just manner) by legal actors are more likely to recognize the authority of these actors (i.e., their legitimacy), cooperate with them, and avoid non-compliant and criminal behaviors (11). Based upon these promising outcomes, shared decision making is being advanced in work with individuals who are under community supervision and who have MI (12–14). Nonetheless, there remain several challenges to employing shared decision making with people who have MI, including practical issues like time constraints during the clinical encounter, insufficient provider training in shared decision making, and limited treatment options [for reviews, see (15, 16)]. While these same challenges are likely present in community corrections settings, the focus here is on three conceptual barriers that exist for the embrace and implementation of shared decision making specific to this post release process. Recommendations—reframing and practice enhancements—are provided that may be utilized to promote shared decision making and promote recovery among justice-involved persons living under community supervision. To reflect recent developments in the field, research and systematic reviews published within the last 5 years were prioritized for coverage in the following narrative review of the literature. Further, while the issues covered may be relevant to employing shared decision making in physical health care decisions, the focus here is on collaboration around engagement in what may be termed social care, social services, and/or behavioral health care.

## Who is the Client—The Public or the Supervisee?

Shared decision making has developed in treatment environments where clarity exists as to who is involved in the shared decision making process (i.e., the provider and the patient, the therapist and the client). These dyads form the basis for much of the research on shared decision making with service users who have a MI (9). However, the following excerpt from Young's 2017 qualitative study with social workers employed in criminal justice settings illustrates how, in the context of community supervision, this provider-consumer dichotomy may be considered less than clear cut:

The court is my client and if I forget that and I treat the participant as my client, then I'm doing something wrong because the court's client is the community and so that's where safety comes in first. And so before my client's needs, I have to look at the court's needs and the need to protect community safety before I get to my client [(17), p. 106].

This blurring of client focus is often referred to as the “dual role” in community corrections, where officers are called upon to facilitate or provide rehabilitative services while also serving as guardians of public safety [for a review, see (18)]. Adherence to this dual-role perspective may function as a hindrance to shared decision making by perpetuating perceptions that evidence-based offender rehabilitation approaches are at odds with, or secondary to, public safety. Such distinctions may have limited utility in modern community supervision.

Yanos et al. provide a thorough history and summary of similarly competing priorities in the mental health service system that they term “community protection vs. individual healing” (19). Using case examples of individuals leveraged into treatment through assisted outpatient treatment and mental health court processes, the authors illustrate how these competing priorities contribute to role confusion among service providers and to distrust of mental health services among consumers. These authors suggest amelioration through greater delineation and differentiation of service missions between law enforcement and mental health providers and greater transparency in service policies and procedures. This role differentiation, when facilitated through partnerships or interprofessional teams of community corrections and mental health and supportive service providers, can reflect the need to holistically address the complex needs of persons with MI under community supervision to advance public health and safety (20). In addition, probation and parole officers who are trained in cognitive behavioral counseling strategies, who understand the importance of attending to the safety and security needs of the supervisee, such as the need for permanent and supportive housing, and who embrace effective community supervision strategies, such as cognitive restructuring (14, 21, 22) also exemplify the reality that there is and can be more unity than duality among treatment and public safety activities in community corrections.

Providing and facilitating access to behavioral health and other rehabilitative and supportive social services to individuals under community supervision with MI should not be viewed as anything other than supportive of public safety. In much the same way that vaccinations further the overall health of the community, provision of treatment services that address criminogenic needs and work to reduce recidivism among individuals under community supervision, enhance community safety (23). This is not to suggest a denial of the potential of supervisees with MI (or even of a vaccine) to be directly harmful under certain circumstances. Expectations for community-based treatment and collaborative decision making need to be reexamined and even suspended during acute periods when the person under supervision is substantially impaired and/or experiencing heightened acuity of an existing mental illness that place themselves or others at risk of harm. However, as discussed in the next section, these circumstances do not often require eschewing person-centered treatment as a standard practice.

Adopting the perspective that the needs of the client to support criminal desistance are essentially the same as the community's needs for public safety highlights the importance of establishing collaborative working relationships that promote supervisee engagement in rehabilitative services. For example, as shared decision making with patients can improve engagement in heath promoting behaviors and treatments, it can also potentially increase the engagement of community supervisees in rehabilitative services (12). Indeed, recent guidance on community corrections supervision highlights competencies that include a focus on development of positive interpersonal relationships among officers and supervisees (21). The potential for these relationships to promote collaboration and to engage individuals in rehabilitative services and enhance public safety is reflected in recent research indicating that bidirectional communication that supports shared decision making among officers and those they supervise contributes to trust, respect, working alliance, and goal agreement, all of which reduce reactance toward the officer and supervisee recidivism (12, 24, 25).

## Can Supervisees be Afforded the Dignity of Risk?

A benefit of recovery-oriented approaches, like shared decision making, that are based upon self-determination is that such services promote opportunities to learn, first-hand, what “works” and what does not work in regard to goal attainment. Empowering others by allowing them this opportunity is referred to as the “dignity of risk” (26). A recent review of the literature on application of this concept by providers of community-based supports for people living with a range of physical and mental challenges indicates substantial awareness of the value and benefits of risk taking (27). However, that review also indicated a tendency among providers for paternalism and hazard avoidance over providing support for positive risk-taking behavior. This tendency toward risk-aversion is likely heightened in community corrections settings, where consequences are perceived to be grave (28). However, as Marsh and Kelly's findings point out, these perceptions are often inflated to the detriment of the individual being scrutinized (27):

Overestimating risk enables staff to justify restricting choices and limiting activities that may be the source of enjoyment for people with mental illness or intellectual disabilities …. Although extreme harm events can and do occur, the types of risks that people face from day-to-day have less severe outcomes (p. 304).

One potential antidote to hypervigilance to risk is the incorporation of strengths into supervision plans. There is a growing interest in strengths and the integration of strength-based elements into risk assessment, accompanied by enhanced awareness that doing so improves predictive accuracy and provides valuable case planning information (29, 30). Knowledge of existing strengths can be incorporated into service planning to ensure that resources are maximized and that certain risks are mitigated. In this way, planning can involve identification of methods for activating strengths toward goal attainment as well as identification of methods to respond to and reduce risky situations and behaviors. For example, if a supervisee identifies as a goal to maintain stable housing but acknowledges that problems with substance use and substance using visitors have impeded attainment of this goal in the past, then planning might involve the identification of existing resources (i.e., strengths) that can help limit substance use (e.g., family or peer supports, community treatment programs). Additionally, planning may focus on the identification of areas where capabilities need to be developed to avoid substance using peers (e.g., assertiveness training, prosocial leisure opportunities).

Indeed, there are myriad ways in which community corrections officers can support shared decision making with those under supervision. Matejkowski et al. (13), describe how community corrections can facilitate compliance with treatment mandates via shared decision making by working with clients to identify mutually agreeable treatment providers and by collaborating with providers and with clients to identify client-centered goals and agreed-upon service planning to attain these goals.

Specifically, this translates into collaborative decision making with the person receiving services about what goals are most important, what approaches are to be taken, and selection of ways of monitoring and self-monitoring the outcomes. Within these processes, the role of the officer is to help supervisees continually examine their thinking and behaviors, communicate and advise about the acceptability of their decisions and when their decisions conflict with public safety goals, and implement measures to prevent criminal behavior and recidivism (p. 615).

In sum, persons with mental illness under community supervision can and should be offered the dignity of risk. These opportunities can be increased through an open and reflective discussion between the officer and the supervisee of the risks associated with any decision, the value of the decision to the supervisee, and shared concerns associated with making that decision (31, 32). Incorporating strengths into the decision making calculus will aid in the development of methods for managing potential challenges so that self-determination can be promoted and mutually agreed upon goals can be attained.

## Can Supervisees With Mental Illness Rationally Contribute to Shared Decision Making?

Among the general public, persons labeled both as an offender and as a person with a mental illness face a dual stigma that can include perceptions of such persons as dangerous, violent, or dishonest (33–35). Among community corrections officers, particularly those who are trained to work with supervisees who have MI, these negative perceptions appear to be less common (36, 37) and to impact risk assessment and case management practices marginally (38). These less discriminatory views and actions may be the result of the intensive nature of community supervision. Specifically, studies have shown that having interpersonal contact with a person with a criminal history is associated with more positive attitudes, perhaps due to an increased sense of homophily between those with and without a conviction (39, 40). These frequent contacts may also provide opportunities for supervisors to witness what has been observed in healthcare, namely persons with MI making rational decisions about their care (41).

Negative attitudes toward people with MI and justice involvement is relatively low among community corrections officers (36, 37) and both supervisors and supervisees have endorsed the use of shared decision making in their work together (12). Nonetheless, stigma can still impose an obstacle to use of shared decision making in this context. For example, a survey aimed at identifying predictors of attitudes supportive of shared decision making among community corrections officers in the United States reported that feelings reflecting stigma toward people with MI had the strongest relationship with attitudes supportive of shared decision making (39). That is, perceiving supervisees with MI as fundamentally different from supervisors or “too sick” to collaborate in supervision planning were both negatively associated with support of shared decision making. That the same survey found familiarity with mental health recovery approaches positively related to support of shared decision making among supervisors suggests potential remedies.

Training that specifically promotes an awareness of fundamental commonalities among supervisees and supervisors and that emphasizes a view of mental illness as a disease that, while sometimes disabling, does not preclude the supervisee from giving input to and participating in decisions, can reduce negative perceptions and social distance toward people with mental illness and promote shared decision making. Anti-stigma interventions, particularly those that involve contact between law enforcement officers and persons who have lived experience with mental illness, have been shown to improve attitudes, behaviors, and mental health literacy among police officers (42). Specific to community corrections, training that provided information on personality and major mental disorders, included guidance on how to talk with probationers about their mental health and medications, and described how to respond to supervisees in a mental health crisis was effective at increasing mental health knowledge and decreasing stigma toward people with MI among probation officers (43).

## Discussion and Conclusion

Shared decision making holds promise as an approach to support persons with MI under supervision in the community. This practice allows supervisees to contribute to their supervision plans, which can promote their engagement with services identified therein and thereby extend their stable community tenure. Employing shared decision making with this population need neither be considered prohibitively risky, nor should risk of supervisee failure be entirely avoided. Indeed, with a solid understanding of recovery, shared goals, and individual strengths, community corrections supervisors can employ shared decision making with supervisees in a way that empowers clients and strengthens communities.

## Author Contributions

JM wrote the manuscript.

## Conflict of Interest

The author declares that the research was conducted in the absence of any commercial or financial relationships that could be construed as a potential conflict of interest.

## Publisher's Note

All claims expressed in this article are solely those of the authors and do not necessarily represent those of their affiliated organizations, or those of the publisher, the editors and the reviewers. Any product that may be evaluated in this article, or claim that may be made by its manufacturer, is not guaranteed or endorsed by the publisher.
